# Implantation of computed tomography-guided high-dose-rate ^192^Ir brachytherapy in oldest old patients with advanced non-small cell lung cancer: A case report and literature review

**DOI:** 10.1097/MD.0000000000032450

**Published:** 2022-12-30

**Authors:** Ran Cui, Xiu-Lan Wang, Jian Cao

**Affiliations:** a Department of Oncology, The First People’s Hospital of Neijiang, Neijiang, Sichuan, China.

**Keywords:** case, chemotherapy, HDR ^192^Ir brachytherapy, NSCLC, oldest old

## Abstract

With the increasing aging and the popularization of medical diagnosis, the growing number of oldest old with lung cancer needs to be focused on. Several medical and physiological challenges often accompanying the oldest old cancer patients make the choice of the optimal treatment daunting. The current research suggests that people who get adequate treatment can benefit, but it is worth discussing which treatment will benefit them more. High-dose-rate (HDR) ^192^Ir brachytherapy deserves attention in this context owing to its association with less trauma and reduced complications.

**Case presentation:**

An 86-years-old woman with a right glandular lung carcinoma presented with progressive lesions 11 months after chemotherapy. Because of her old age and poor performance status (eastern cooperative oncology group performance status 3), she received HDR ^192^Ir brachytherapy for her right lung lesion without any common complications, such as pneumothorax and hemorrhage. She continued on 0.25 g oral gefitinib each day after received brachytherapy treatment. The right lung lesion keeps a partial response until 18 months later now.

**Conclusion:**

HDR ^192^Ir brachytherapy can potentially be used as a safe and effective choice for the oldest old with advanced non-small cell lung cancer. It can especially benefit cancer patients with concurrent chemotherapy or targeted therapy.

## 1. Introduction

Lung cancer is the most commonly occurring cancer globally and is considered the primary type for cancer-related deaths worldwide.^[1]^ However, during diagnosis, most of the patients suffer from metastatic disease. A systemic, palliative treatment can be regarded as a crucial therapeutic option. Over 50% of advanced non-small cell lung cancer (NSCLC) are aged ≥ 65 years old.^[2]^ The medical and physiological challenges often accompanying the oldest old cancer patients make the selection of their optimal treatment daunting.^[3,4]^ Therefore, most prospective oncological studies primarily contain younger patients with good performance status. Thus, there is limited information regarding optimal cancer treatment in older patients. Yang et al explored the national cancer data base from 2004 to 2014 to evaluate the survival of patients aged ≥ 90 years. They reported that 57.6% of old NSCLC patients did not receive any therapy. Further, those receiving any treatment exhibited markedly better survival compared to patients who received no therapy (5-year survival, 9.3% [95% confidence interval, 8.0–10.7%] vs 1.7% [95% confidence interval, 1.2–2.2%]).^[5]^ Similarly, Schulkes et al found that the oldest old NSCLC patients receivingstandard treatment exhibited longer survival than those don’t get any treatment.^[6]^ Therefore, the treatment for the oldest old NSCLC patients warrants attention.

Minimally invasive high-dose-rate (HDR) ^192^Ir brachytherapy is often used in combination with conventional radiotherapy or as an option for palliative treatment clinically. The oldest old exhibit more comorbidities and worse functional status than the younger ones. Thus, the oldest old are less likely to undergo surgery or radiotherapy, especially those with advanced NSCLC.^[7]^ In such patients, HDR ^192^Ir brachytherapy is a safe, useful, less-complicated, and valuable solution. For most of the oldest old with advanced NSCLC, especially those with comorbidities and poor functional status, treatment strategies with fewer complications are more suitable. The advantages of HDR ^192^Ir brachytherapy over other interventional procedures result from its accurate dosage measurement and fewer complications. HDR ^192^Ir Brachytherapy can possibly be provided as the practical alternative treatment approach for the oldest old with advanced NSCLC.

In the current study, we reported a clinical practice adopted for an oldest old with advanced NSCLC treated with HDR ^192^Ir brachytherapy. For our approach, a good curative effect was observed.

## 2. Case report

**Figure 1.**
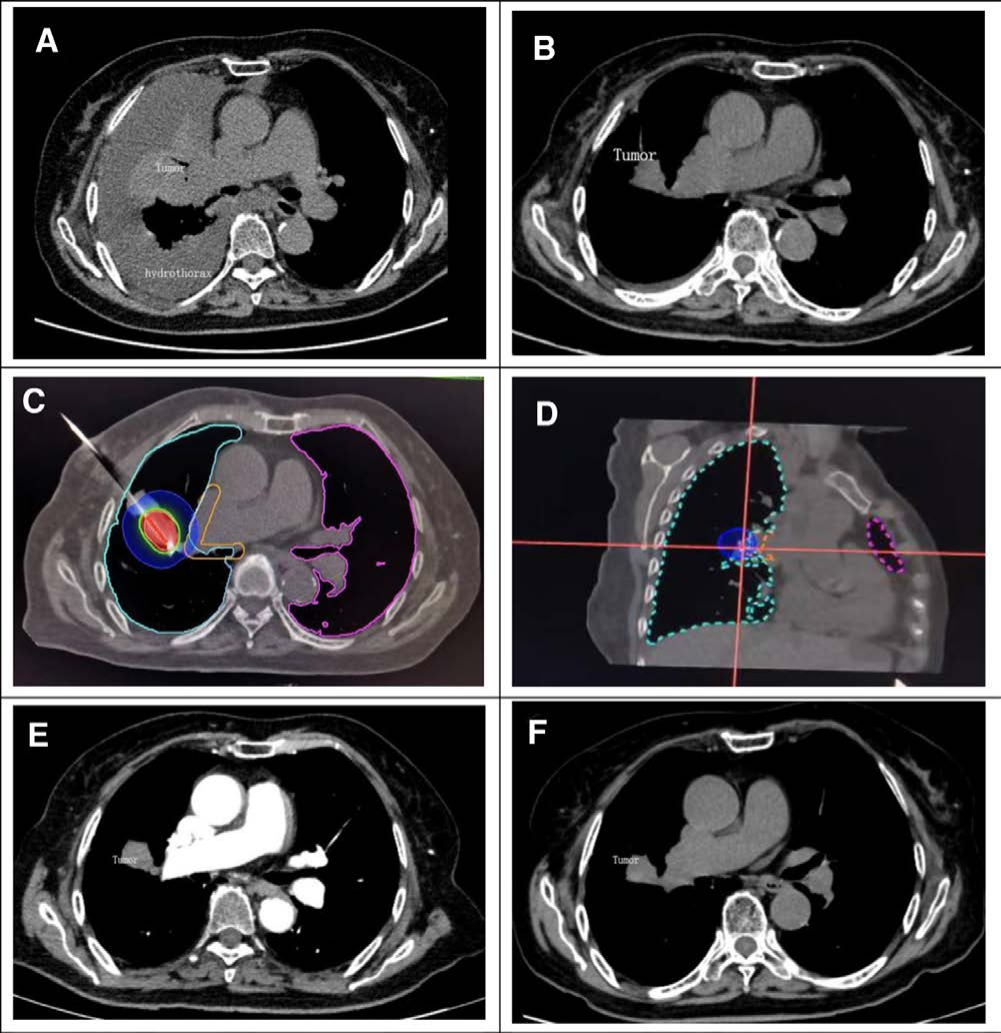
Changes observed in the patient before and after receiving HDR ^192^Ir brachytherapy. (A): CT at the time of admission revealed a 4.8 × 4.2 cm right thoracic tumor lesion with a large amount of pleural effusion; (B): Only a small amount of pleural effusion was left before HDR ^192^Ir brachytherapy; (C and D): A single dose of 27 Gy to the target lesion; (E): After 2 weeks post-treatment, the size of the lesion was reduced to 3.0 × 2.3 cm; (F): After 18 months of treatment, the lesion showed no progression. Gy = gray, HDR = high-dose-rate.

An 86-years-old woman with right glandular lung carcinoma was referred to our hospital due to gradually aggravating dyspnea. Her medical history included moderate-to-severe chronic obstructive pulmonary disease. In addition, 11 months ago, she had a CT -guided needle biopsy of the lung. She was diagnosed with advanced glandular lung carcinoma (cT2bN1M0, UICC 8^th^) with EGFR mutation. The patient intravenously received a TP regimen (Paclitaxel 200 mg/m^2^ d1 + Cisplatin 100 mg/m^2^). After 2 cycles of chemotherapy, complications such as bone marrow suppression developed. Thus, chemotherapy was discontinued. She then continued oral gefitinib 0.25 g each day. She then developed back pain, and her CT scan revealed a right lung lesion with pleural effusion and 8^th^ thoracic vertebra bone metastases (Fig. 1A). Because of her old age and poor performance status 3, we decided to consult the feasibility of CT-guided brachytherapy in the radiation oncology department. After aspiration of pleural effusion and necessary nutritional supplementation (Fig. 1B), she received HDR ^192^Ir brachytherapy to her right lung lesions at a total dose of 27 Gray (Gy) (Fig. 1C, D). There were no common complications, such as pneumothorax and hemorrhage. Two weeks later, a CT scan of her chest was performed, which revealed that the right lesion was smaller than before(Fig. 1E). Subsequently, she returned home, continued oral gefitinib 0.25 g each day, and kept coming to the hospital for review every month. After 18 months of follow-up, there was still no progression of her pulmonary and thoracic lesions (Fig. 1F).

## 3. Discussion

Demographic changes in the current society are expected to increase the number of oldest old with lung cancer in the coming decades.^[8]^ Chemoradiotherapy may effectively prolong the overall survival (OS) as well as enhance the quality of life.^[9]^ However, the ongoing research shows that lung cancer patients aged ≥ 80 years, when compared with younger patients, are less likely to receive surgery or radiotherapy, thus presenting poorer outcomes.^[10]^ This practice is primarily attributed to the unwillingness of the oldest old to receive treatment, more likely due to comorbidities and unfavorable functional status. Therefore, finding an effective solution that suits the oldest old is worth exploring. Based on the current case, for the oldest old with advanced NSCLC, especially those who have received chemotherapy, HDR ^192^Ir brachytherapy might prove to be a good treatment modality to control the progress of the tumor and effectively prolong the patient OS.

The application of brachytherapy can lead to the efficient inhibition of tumor cell proliferation while sparing the surrounding normal tissues due to exposure to considerably lower radiation (< 25%) compared to the tumor cells.^[11]^ Additionally, HDR radiation delivered by ^192^Ir enables the cells to activate their DNA damage response less efficiently than after constant irradiation.^[12]^ CT-guided ^192^Ir brachytherapy has been extensively applied as an efficient and minimally invasive therapy for various tumors, especially NSCLC.^[13–17]^ A retrospective analysis showed that HDR ^192^Ir brachytherapy could provide safe and efficient treatment for lung cancer patients, with a complete response plus partial response rate for gross lung tumor volume  < 5 cm at 6 months reaching 100%. Moreover, with this approach, the local control rate for gross lung tumor volume at 1 year reached 96.9%. When it comes to organs at risk, D1000 cm^3^ and D1500 cm^3^ for lung in 1, 3, and 5 fractions of patients subjected to HDR ^192^Ir brachytherapy did not show any significant difference from the patients who received stereotactic body radiotherapy (all *P* > .05). Meanwhile, the remaining dosimetric parameters were found to be significantly lower in patients undergoing HDR ^192^Ir brachytherapy than those receiving stereotactic body radiotherapy (all *P* < .01).^[18]^ HDR ^192^Ir brachytherapy can be considered an effective alternative treatment modality for NSCLC patients.

Although HDR ^192^Ir brachytherapy is a less invasive treatment compared to other interventional procedures, complications still occur frequently, the most common being pneumothorax and bleeding. In this study, the patient developed no complications. Additionally, the success of HDR ^192^Ir brachytherapy relies on the accurate placement of radioactive seeds in the tumor volume by careful treatment planning. We used CT images to guide the implantation and planned for dose optimization. In addition, seed positions decided to offer appropriate dose distribution containing minimum peripheral dose coverage as well as the protection of surrounding tissues, such as trauma to the esophagus, bronchia, and vessels, based on the available methods for calculating optimal seed locations. Clinical experience is required to complete this series of operations perfectly, which may be 1 of the reasons why this technology could not be popularized among young doctors. Dou et al designed a robotic system for lung cancer brachytherapy, which was able to locate the template with clinically acceptable accuracy under the CT environment, overcoming the aforementioned problem. They reported that the robot was CT-compatible and responded dependably to the control commands. Besides, the mean registration accuracy of the robotic system reached 0.49 ± 0.29 mm. Based on phantom experiments, the accuracy of needle insertion reached 1.5 ± 1.7 mm at a depth range of 30 to 80 mm. In addition, the time taken by the robotic system to adjust the template to the target position was 12 minutes on average. Moreover, relative to the manual positioning procedure in phantom experiments, the robotic system was able to save approximately 30 minutes in the overall procedure.^[19]^

When the biologically effective dose is more than 100 Gy in external beam radiotherapy, it is considered to be an ablative dose.^[20]^ A significant advantage of HDR ^192^Ir brachytherapy against external radiotherapy is the delivery of a higher dose to the tumor, which is associated with a better local control rate. In the current work, we prescribed a single dose of 27 Gy to the target lesion (with a median size of 4.8 cm), with the anticipated biologically equivalent dose of around 120 Gy for conventional external radiation. The tumor was well locally controlled, and there were no serious complications. According to Skowronek et al, when the biologically equivalent dose is reached, any larger dose of radiotherapy cannot benefit the patients.^[21]^ Therefore, selecting a smaller radiotherapy dose that meets the ablative dose may make the treatment effective while minimizing the associated risks of overdose.

Brachytherapy is an effective method to control tumors when the patients cannot tolerate surgery and external beam radiotherapy, but it is limited to controlling the progress of the tumor locally. There is no therapeutic effect on systemic symptoms and metastases. So, when this approach is combined with chemotherapy or targeted therapy, a better curative effect may be obtained. Recently, Zhang et al showed that HDR ^192^Ir brachytherapy, combined with concurrent or sequential chemotherapy, is safe and efficient for peripheral locally advanced NSCLC, with overall response rates (complete and partial) for the primary mass and positive lymph nodes being 100% and 92.3%, respectively. In addition, with the median OS of 22.5 months, the 1-year and 2-year OS rates reached 90.9% and 67%, respectively. And no severe complications occurred during the follow-up.^[22]^ So, we believe that HDR ^192^Ir brachytherapy combined with chemotherapy or targeted therapy may be an alternative therapeutic modality that could provide a remarkable improvement in the outcome of NSCLC patients, especially the oldest old.

In conclusion, HDR ^192^Ir brachytherapy provided a safe and effective choice for the oldest old with advanced NSCLC, especially those who received concurrent chemotherapy or targeted therapy.

## Author contributions

**Formal analysis:** Ran Cui.

**Writing – original draft:** Xiu-Lan Wang.

**Writing – review & editing:** Jian Cao.

## References

[1] Sung H Ferlay J Siegel RL. Global cancer statistics 2020: GLOBOCAN estimates of incidence and mortality worldwide for 36 cancers in 185 countries. CA Cancer J Clin. 2021;71:209–49. 33538338 10.3322/caac.21660

[2] Gridelli C Langer C Maione P. Lung cancer in the elderly. J Clin Oncol. 2007;25:1898–907. 17488989 10.1200/JCO.2006.10.3085

[3] Repetto L Balducci L. A case for geriatric oncology. Lancet Oncol. 2002;3:289–97. 12067806 10.1016/s1470-2045(02)00730-1

[4] Repetto L Venturino A Fratino L. Geriatric oncology: a clinical approach to the older patient with cancer. Eur J Cancer. 2003;39:870–80. 12706355 10.1016/s0959-8049(03)00062-5

[5] Yang C-FJ Brown AB Deng JZ. The oldest old: a National Analysis of outcomes for patients 90 years or older with lung Cancer. Ann Thorac Surg. 2020;109:350–7. 31757356 10.1016/j.athoracsur.2019.09.027

[6] Schulkes KJG Pouw CAM Driessen EJM. Lung cancer in the oldest old: a nation-wide study in the netherlands. Lung. 2017;195:627–34. 28631153 10.1007/s00408-017-0026-1

[7] Soror T Kovács G Wecker S. Palliative treatment with high-dose-rate endobronchial interventional radiotherapy (Brachytherapy) for lung cancer patients. Brachytherapy. 2021;20:1269–75. 34429246 10.1016/j.brachy.2021.06.149

[8] Howlader N Noone AM Krapcho M. SEER cancer statistics review, 1975-2017. 2020; based on November 2019 SEER data submission. Available at: https://seer.cancer.gov/csr/1975_2017/.

[9] Atagi S Kawahara M Yokoyama A. Thoracic radiotherapy with or without daily low-dose carboplatin in elderly patients with non-small-cell lung cancer: a randomised, controlled, phase 3 trial by the Japan Clinical Oncology Group (JCOG0301). Lancet Oncol. 2012;13:671–8. 22622008 10.1016/S1470-2045(12)70139-0

[10] Owonikoko TK Ragin CC Belani CP. Lung cancer in elderly patients: an analysis of the surveillance, epidemiology, and end results database. J Clin Oncol. 2007;25:5570–7. 18065729 10.1200/JCO.2007.12.5435

[11] Lu M Pu D Zhang W. Trans‑bronchoscopy with implantation of 125I radioactive seeds in patients with pulmonary atelectasis induced by lung cancer. Oncol Lett. 2015;10:216–22. 26171002 10.3892/ol.2015.3204 PMC4487145

[12] Veigel C Hartmann GH Fritz P. Dedicated high dose rate 192Ir brachytherapy radiation fields for in vitro cell exposures at variable source-target cell distances: killing of mammalian cells depends on temporal dose rate fluctuation. Phys Med Biol. 2017;62:1613–31. 28145285 10.1088/1361-6560/aa587c

[13] Poon E Verhaegen F. Development of a scatter correction technique and its application to HDR multicatheter breast brachytherapy. Med Phys. 2009;36:3703–13. 19746803 10.1118/1.3157105

[14] Imamura F Ueno K Kusunoki Y. High-dose-rate brachytherapy for small-sized peripherally located lung cancer. Strahlenther Onkol. 2006;182:703–7. 17149576 10.1007/s00066-006-1536-6

[15] Schraube P Fritz P Becker HD. Ergebnisse der endoluminalen high-dose-rate-bestrahlung von zentralen nichtkleinzelligen bronchialkarzinomen. Strahlenther Onkol. 1993;169:228–34. 8387698

[16] Micke O Prott FJ Schäfer U. Brachytherapie in der palliativ-behandlung von patienten mit rezidiv eines nicht-kleinzelligen bronchialkarzinoms nach vorausgegangener strahlentherapie. Strahlenther Onkol. 1995;171:554–9. 8571174

[17] Hauswald H Stoiber E Rochet N. Treatment of recurrent bronchial carcinoma: the role of high-dose-rate endoluminal brachytherapy. Int J Radiat Oncol Biol Phys. 2010;77:373–7. 19836162 10.1016/j.ijrobp.2009.05.041

[18] Pang H Wu K Shi X. Hypofractionated 192Ir source stereotactic ablative brachytherapy with coplanar template assistance in the primary treatment of peripheral lung cancer. J Contemp Brachyther. 2019;11:370–8. 10.5114/jcb.2019.87218 PMC6737569 31523239

[19] Dou H Jiang S Yang Z. Design and validation of a CT‐guided robotic system for lung cancer brachytherapy. Med Phys. 2017;44:4828–37. 28657112 10.1002/mp.12435

[20] Chang JY Cox JD. Improving radiation conformality in the treatment of non–small-cell lung cancer. Semin Radiat Oncol. 2010;20:171–7. 20652085 10.1016/j.semradonc.2010.01.005 PMC2905742

[21] Skowronek J Kubaszewska M Kanikowski M. HDR endobronchial brachytherapy (HDRBT) in the management of advanced lung cancer–comparison of two different dose schedules. Radiother Oncol. 2009;93:436–40. 19854525 10.1016/j.radonc.2009.09.005

[22] Xiang L Zhang JW Lin S. Computed tomography–guided interstitial high-dose-rate brachytherapy in combination with regional positive lymph node intensity-modulated radiation therapy in locally advanced peripheral non–small cell lung cancer: a phase 1 clinical trial. Int J Radiat Oncol Biol Phys. 2015;92:1027–34. 26194678 10.1016/j.ijrobp.2015.04.019

